# The Deconvolution of Human Hematopoiesis

**DOI:** 10.1097/HS9.0000000000000153

**Published:** 2018-11-29

**Authors:** Michael D. Milsom

**Affiliations:** 1 Division of Experimental Hematology, German Cancer Research Center (DKFZ), Heidelberg, Germany; 2 Heidelberg Institute for Stem Cell Technology and Experimental Medicine (HI-STEM), Heidelberg, Germany

The human hematopoietic system is comprised of at least 18 different individual mature cell types, the majority of which are thought to be derived from multipotent hematopoietic stem cells (HSCs). Over many decades, hierarchical maps of the hematopoietic system have evolved, based on the work of many different groups that have employed a range of different in vitro and in vivo assays, using both hematopoietic cells from human donors and those from model organisms. Such maps describe how HSCs give rise to different blood cell lineages via a series of sequential commitment steps, where cells transition through various defined progenitor populations. Far from being just an academic pursuit, these hierarchical structures formed a framework upon which the field could attempt to gain insight into the cellular and molecular basis of processes such as malignant transformation, leukemic disease propagation via so-called leukemic stem cells and bone marrow failure. Recent technological advances that have facilitated large scale analysis of molecular signatures within single hematopoietic cells, have generated data that have challenged such models by inferring that hematopoiesis can be best described by a continuous process of differentiation, as opposed to the canonical view of discrete differentiation steps (reviewed in Ref.^1^). In the mouse model of hematopoiesis, several approaches have been used to mark individual hematopoietic cells with unique barcodes, thus facilitating the definitive identification of cells that have a common ancestry and providing fresh new insights into how native hematopoiesis is sustained.^2,3^ However, what has been sorely missing has been an analogous approach to interrogate clonal relationships within the native human hematopoietic system. In a recent edition of Nature, the groups of Tony Green, David Kent and Peter Campbell have taken advantage of spontaneously occurring mutations in order to map hierarchical relationships between HSCs and their progeny within a healthy 59-year-old man.^4^

**Figure 1:**
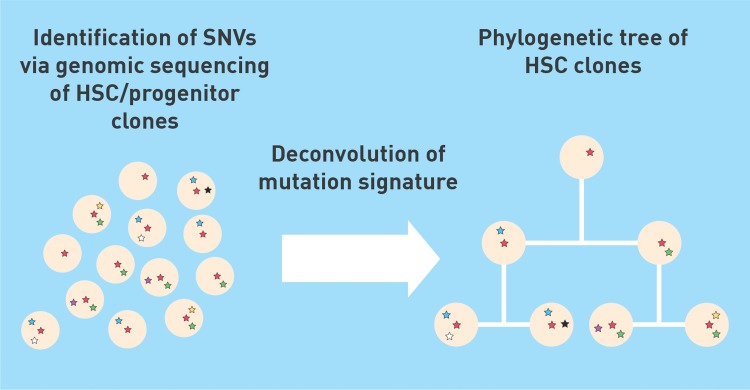
The pattern of spontaneous somatic mutations that are sequentially acquired within different HSC clones following division (represented by different colored stars) can be deconvoluted to generate a phylogenetic tree.

In order to identify single nucleotide variants (SNVs) within the genome of HSCs that may act as unique barcodes for a given cell, the authors first isolated single HSCs/progenitors from the bone marrow and blood of the donor and then clonally expanded each cell in vitro. The resulting colonies were then individually subjected to whole genome sequencing and, by comparing to the germline sequence, SNVs could be identified that were present in every genome within the colony, and therefore must have been present in the original clone. Using this approach, genome-wide SNVs were characterized in 140 individual HSCs/progenitors. Of the almost 130,000 SNVs characterized, nearly 9000 could be found in more than one clone, while the remainder were unique. The authors were then able to reconstruct a phylogenetic tree encompassing these HSCs, based on the concept that shared mutations allowed the identification of direct lines of descent while unique mutations facilitated the identification of branch points in the hierarchy (Fig. 1). Intriguingly, this phylogenetic tree provided a genealogy for each cell that had a mutation record going back to the very first cell divisions following conception of the donor. Indeed, 2 specific mutations could be identified that were mutually exclusive across all clones analyzed, but with every cell analyzed possessing one of these mutations. Almost the exact same pattern of inheritance was observed when cells descended from the ectoderm germ layer were analyzed from tissue sampled in a buccal swab. This common pattern of inheritance suggests that these mutations were acquired very early during embryonic development, certainly before gastrulation results in the segregation of mesoderm and ectoderm, and likely in one of the first cell divisions postfertilization. Thus, early branching points in the phylogenetic tree represent mutations acquired during development, while later branch points could be attributed to mutation events that took place within the hematopoietic lineage itself.

By identifying mutation branch points that likely occurred within the HSC pool and then persisted in long-lived HSC progeny, the authors could surmise that self-renewal divisions had taken place. The authors then modeled how the total size of the HSC pool altered during the lifetime of the individual, by estimating the time interval between branch points of the clones analyzed. As expected, the HSC pool size went through a period of rapid expansion up until late childhood, but then remained relatively stable throughout adulthood. This latter finding does not appear to correlate with findings within the mouse model of hematopoiesis, where HSC numbers dramatically increase during aging. However, it could be that such population changes within humans take place at later time points.

A targeted resequencing approach was then performed to assess how the SNVs detected within the sampled HSC/progenitor cells were distributed in mature peripheral blood cells. Interestingly, the majority of these SNVs could not be detected within circulating granulocytes, suggesting that the size of the HSC pool that actively contributes to granulopoiesis dramatically exceeds the sample size. Indeed, the authors could estimate that in the order of tens to hundreds of thousands of HSCs regularly contribute to granulocyte production at any one time. However, the analysis also uncovered that some branches of the HSC pool were disproportionately contributing more mature cells than others, which may relate to the concept of dormant and active HSCs that has been documented in the murine system using label-retention assays. In order to assess the multipotency of HSCs, the same panel of SNVs was analyzed in peripheral T and B cells, then compared with the data acquired in granulocytes. Mutations that were defining of early branch points in the hierarchy could be detected in all 3 mature blood cell populations, suggesting that at some point during development, common multipotent ancestors existed for all 3 cells types. When mutations were considered that occurred in the plateau phase of HSC pool size, during adulthood, approximately 1/3 of all the branches that could be analyzed contributed to the production of more than one cell lineage, formally demonstrating multipotency of adult human HSCs under native conditions. Interestingly, some adult HSCs were found to contribute to active production of granulocytes and B cells, but T cell progeny could not be detected. This observation may represent a limitation imposed by sample size, but could also be representative of either lineage bias within the human HSC pool, or differential kinetics of replenishment of the T cell lineage as compared to B cells and granulocytes.

Taken together, this study represents an intriguing proof of concept that one can directly interrogate the genealogy of cell lineages within the human hematopoietic system using endogenously occurring mutations. While current limitations on sequencing cost and acquisition of appropriate samples will restrict the widespread application of this approach in the near future, the possibilities for extending this concept to the dissection of hematologic disease are very exciting.
